# The Association between Self-Reported Grocery Store Access, Fruit and Vegetable Intake, Sugar-Sweetened Beverage Consumption, and Obesity in a Racially Diverse, Low-Income Population

**DOI:** 10.3389/fpubh.2014.00229

**Published:** 2014-11-11

**Authors:** Lauren Nichol Gase, Amelia Rose DeFosset, Lisa V. Smith, Tony Kuo

**Affiliations:** ^1^Division of Chronic Disease and Injury Prevention, Los Angeles County Department of Public Health, Los Angeles, CA, USA; ^2^Office of Health Assessment and Epidemiology, Los Angeles County Department of Public Health, Los Angeles, CA, USA; ^3^Department of Family Medicine, David Geffen School of Medicine at the University of California Los Angeles, Los Angeles, CA, USA; ^4^Department of Epidemiology, Los Angeles Fielding School of Public Health, University of California Los Angeles, Los Angeles, CA, USA

**Keywords:** obesity, food dessert, fruit and vegetable consumption, food access, food environment

## Abstract

This study sought to examine the relationship between self-reported time and distance to the nearest retail grocery store, healthy and unhealthy food consumption, and objectively measured body mass index (BMI). We conducted a survey with 1,503 racially diverse, low-income residents at five public health centers in Los Angeles County. Most participants reported shopping at a supermarket (86.7%) and driving (59.9%) to their usual source for groceries. Over half reported living less than a mile from (58.9%) and traveling 5 min or less to reach (50.3%) the nearest grocery store. In the multivariable regression models, neither self-reported distance nor time to the nearest grocery store was consistently associated with fruit and vegetable intake, sugar-sweetened beverage consumption, or BMI. Results suggest that the need to consider access and quality as well as urban planning and transportation, when examining the relationship between the retail food environment and health outcomes.

## Introduction

Poor diet is associated with a variety of health problems, including obesity, cardiovascular disease, hypertension, diabetes, osteoporosis, and some forms of cancer (1). In 2011–2012, more than one-third of all adults were classified as obese in the U.S. (2). Obesity rates are higher among African-Americans (47.8%) and Hispanics (42.5%) than in whites (32.6%) and Asians (10.8%) (2). Estimates suggest that obesity is responsible for $147 billion in annual medical costs (3).

Healthy eating is a complex phenomenon, influenced by a variety of interacting environmental, social, and individual factors (4, 5). For example, environmental effects can be moderated or mediated by demographic, psychosocial, or perceived environment variables (4). While the concept of “food environment” includes multiple dimensions (e.g., access, availability, price, and promotion) and settings (e.g., homes, restaurants, worksites, and recreational facilities), retail food stores are thought to be important contributors to eating patterns because of the frequency and volume at which people purchase food at such locations (4, 6). Among retail stores, supermarkets have been shown to offer the greatest variety of food at the lowest cost (7, 8). In recent years, increased attention has been placed on eliminating the so called “food desert” areas (6). The United States Department of Agriculture (USDA) defines food deserts as existing in communities that are (a) low-income, based on having a poverty rate of 20% or greater or a median family income at or below 80% of the area median family income and (b) low-access, based on the determination that at least 500 individuals or at least 33% of the census tract’s population live more than 1 mile from a supermarket or large grocery store (10 miles, in non-metropolitan census tracts) (9).

Despite the increased attention and funding to eliminate food deserts, much remains unknown about their influence on healthy eating and obesity. Several studies have found associations between access to supermarkets and healthier diets (4). For instance, one study documented a 32% increase in fruit and vegetable consumption among African-American residents per additional supermarket in their census tract (10). However, in a systematic review, few studies found an association between access to supermarkets and fruit and vegetable consumption, although access to supermarkets was associated with lower weight status (11). These null findings are echoed by a number of recent studies (12, 13). For example, studies that have considered price, in addition to proximity, found no association between distance to grocery stores and diet or body mass index (BMI) (14, 15). Notably, at least one study found a negative effect, wherein greater access to grocery stores was associated with higher BMI in women (16).

The purpose of this study was to examine the relationship between self-reported food environment, healthy eating habits, and obesity. The study assessed data on food-purchasing patterns, proximity to food outlets, healthy and unhealthy food consumption, and obesity in a sample of low-income Los Angeles County residents. This study adds to the literature by assessing both self-reported time and distance to the nearest grocery store and examines the association between grocery store access with both dietary and anthropometric outcomes.

## Materials and Methods

### Sample

We analyzed data from the second round of the Los Angeles County Health and Nutrition Examination Survey (LA HANES) conducted during February through April of 2012. The LA HANES was a community health assessment project, which described the health profiles of a sample of low-income community-dwelling adults who utilized multi-purpose public health centers in the region. Health centers operated by the Los Angeles County Department of Public Health typically provide a range of services, including immunizations, treatment of tuberculosis and sexually transmitted diseases, and outreach programs for the surrounding communities. Five health centers were randomly selected from among the pool of centers (*n* = 7) in a pre-defined geographic areas that served a high volume of low-income clients and were the focus area for an array of obesity prevention interventions implemented by several recent federal initiatives such as the *Communities Putting Prevention to Work* program, the Community Transformation Grants, and the Nutrition, Education, and Obesity Prevention Project. The referent profile of these health centers is illustrated by a 2012 survey of clinic visitors, which showed that health center clients were mostly Hispanic (40%) or African-American (22%); uninsured (62%); a number of them unemployed (42%); and nearly half with an annual household income of <$20,000.

### Study protocol

To recruit and enroll participants in LA HANES, trained recruiters approached all individuals waiting for services in the clinic waiting areas to screen them for interest and eligibility. To be eligible, participants had to be (a) age 18 years or older, (b) seeking services at the clinic on the recruitment day, (c) residents of LAC, (d) able to complete surveys in English or Spanish, and (d) not currently pregnant. If an individual was eligible and interested in participating, he/she was scheduled for an appointment.

All participants were asked to return on a Saturday to a designated survey site (i.e., one of five health centers) and completed the full assessment that day including a 95-item questionnaire, which collected information on demographics, tobacco use, and second hand smoke exposure, chronic conditions, sexual health, diet, and physical activity. Trained LA HANES clinical staff collected anthropometric and clinical measures, including height (inches), weight (pounds), and blood pressure. On average, each survey appointment lasted 45 min and each participant was compensated with a $50 Visa gift card for his/her time. The study was approved by the Los Angeles County Department of Public Health Institutional Review Board. The study conformed to all ethical standards, including obtaining written consent from all participants prior to enrollment.

### Measures

#### Weight status

Participant height and weight were each measured two times using a wall-mounted stadiometer (Seca 213) and a digital scale (Seca 876), respectively. These measurements were averaged and used to calculate BMI using the formula – weight (lbs)/[height (inches)] 2 × 703. BMI was treated as a continuous outcome as well as a categorical outcome, with categories determined according to the World Health Organization criteria, classifying participants as underweight/normal ( <24.9), overweight (between 25.0 and 29.9), or obese ( ≥30.0).

#### Healthy eating

Healthy eating was operationalized two ways: (a) fruit and vegetable intake and (b) sugar-sweetened beverage consumption. First, participants were asked to respond to six items about their frequency of eating fruits and vegetables for morning meal/snack, lunchtime/afternoon snack, and suppertime/evening snack for the past 7 days. Participant responses for each of the six items were recoded to correspond to the average number of fruits and vegetables eaten per day – 0 (never), 0.286 (1–3 times per week), 0.714 (4–6 times per week), 1 (1 time per day), 2 (2 times per day), 3 (3 times per day), or 4 (4 times per day) – and summed, using scoring criteria defined by the National Cancer Institute (17). Fruit and vegetable intake was used as both a continuous outcome and a categorical outcome, by classifying participants as eating ≥5 servings or <5 servings per day.

To measure sugar-sweetened beverage (SSB) consumption, participants were asked to report intake of a can, bottle, or glass of (a) soda or pop, (b) sports drinks, (c) energy drinks, and (d) other sugar-sweetened beverages (e.g., lemonade, sweetened tea). Participant responses for each of the 4 items were coded as 0 (never), 2 (1–3 times), 5 (4–6 times), 7 (1 time per day), 14 (2 times per day), 21 (3 times per day), or 28 (4 times per day) and summed to obtain a measure of the average number of SSBs consumed per week (18). Responses were also transformed into a categorical variable, based on whether participants consumed SSBs never (0), weekly (1–6), or daily ( ≥7).

#### Self-reported food environment

To assess shopping patterns, participants were asked “Where do you usually shop for groceries?” Response options included supermarket, corner store, farmer’s market, or other. When stores listed as “other” could be classified into the existing categories, they were recoded. Participants were asked how they usually get from their home to the nearest grocery store. Response options included drive alone, carpool, bus, metro, motorcycle, bicycle, or other. Response options were coded as drive (drive alone, motorcycle, or carpool), public transportation (bus or metro), bicycle/skateboard, walk, or multiple modes (if participants selected >1 response option).

To assess distance from the nearest grocery store, participants were asked to report: (a) how many minutes it usually takes for them to get to the nearest grocery store and (b) how many miles it is from their home to the nearest grocery store (one-way). Variables were treated both as continuous and categorical predictors. Minutes to the grocery store were categorized as ≥5, 6–10, 11–20, <20 min according to the distribution of responses. Distance to the grocery store was categorized as more or <1 mile, according to the “low-access” criteria proposed by the USDA, as well as more or less than 0.5 miles, according to generally accepted distances for walking nationally and internationally (6).

#### Control variables

Participants were asked to provide their age (as a continuous variable), gender (male or female), education level (completed less than high school, high-school graduate/GED, some college, or college graduate/professional degree), and race/ethnicity (African-American, Hispanic, white, Asian, Native American, or multiethnic). Because of the small number of Asians, Native Americans, and those who identified themselves as multiethnic, these race categories were combined into an “other” category.

### Data analysis

Bivariate analyses were conducted to examine the relationship between time to nearest grocery store and (a) mode of transportation used and (b) type of store most frequently shopped at. Multivariable regression analyses were conducted to examine the association between self-reported food environment and diet/obesity. The impact of self-reported food environment was tested using (a) reported distance to grocery stores (as a continuous and categorical variable) and (b) reported time to the nearest grocery store (as a continuous and categorical variable). Outcomes included fruit and vegetable intake, SSB consumption, and BMI. Linear regression was conducted to examine the relationship between food environments and continuous normally distributed outcomes (BMI) and negative binomial regression was conducted for count data variables that displayed a skewed distribution (fruit and vegetable intake and SSB intake) to allow for over-dispersion (19). Negative binomial modeling was used instead of log-transformed linear regression in order to model the dispersion and prevent loss of data (19). Negative binomial regression coefficients were exponentiated to produce rate ratios (vegetables consumed per day), in accordance with standard practices for analysis of cross-sectional count data (19).

To test the robustness of the model, analyses were also conducted using categorical versions of the outcome variables, including logistic regression for whether participants who reported eating ≥5 servings of fruit and vegetables a day and an ordered logistic regression for categories of SSB consumption and BMI. In addition, stratified versions of all multivariable models were constructed to examine differences in the relationship between distance/time to the nearest grocery store and each of the outcomes, by travel mode [drive, public transportation, active transportation (walk, bicycle, or skateboard), or multiple modes].

Case wise deletion was conducted so that all descriptive and multivariable analyses were performed using the sub-set of cases that included no missing data on any of the variables included in the analyses. To examine the impact of missing data, all models were also performed after conducting multiple imputations of missing data with predictive mean matching, using 20 replicates. All multivariable models included demographic control variables (age, gender, race/ethnicity, and education level). Consideration was given to including other potential variables that could confound the relationship between the predictors and the outcomes (e.g., physical activity, employment status, U.S. versus foreign born, other health behaviors). However, the robust null results of multivariable and stratified analyses suggested that further analyses of these confounders were not needed. Because of the use of multiple models, parameter estimates were judged to be significant if the two-tailed *p*-value was <0.01. All analyses were performed using the Stata statistical computing software, version 13.0 (StataCorp LP, College Station, TX, USA).

## Results

In total, 3,317 people were approached in the health centers: 2,184 were eligible and made appointments. Of the 2,184 people invited to participate, 1,503 completed the survey, for a response rate of 69%. Case wise deletion yielded a sample of 1,318 participants.

A majority of the participants were either African-American or Latino. About a quarter reported having a college degree (23.5%), while roughly a third reported being currently employed full or part time (37.3%). Most reported being between the ages of 25 and 44, but ages ranged from 18 to 84 (Table 1). Analysis of participant zip codes suggested that the sample included individuals from a wide geographic range in Los Angeles County.

**Table 1:** **Participant characteristics, round two of the Los Angeles County Health and Nutrition Examination Survey, 2012 (*N* = 1,318)^a^**.

	Number (%) or mean (SD)
**Demographics**
Gender
Female	686 (52.1%)
Male	632 (48.0%)
Race/ethnicity
African-American	649 (49.2%)
Latino	351 (26.6%)
White	167 (12.7%)
Other	151 (11.5%)
Education
Less than high school	204 (15.5%)
High-school graduate	295 (22.4%)
Some college	510 (38.7%)
College graduate	238 (18.1%)
Postgraduate/professional degree	71 (5.4%)
Employment
Employed (full or part time)	492 (37.3%)
Born in the United States	967 (73.5%)
Age (years)	35.6 (12.5)
**Shopping patterns and food access**
Usual grocery source
Supermarket	1142 (86.7%)
Corner store	42 (3.2%)
Farmer’s market	36 (2.7%)
Multiple places	93 (7.1%)
Other	5 (0.4%)
Usual travel mode to grocery store
Car	789 (59.9%)
Public transportation	142 (10.8%)
Walk	238 (18.1%)
Bicycle/skateboard	23 (1.8%)
Multiple modes	126 (9.6%)
Miles from home to nearest grocery store	2.5 (4.1)
Travel time to nearest grocery store (minutes)	10.2 (10.6)
**Eating patterns and body mass index**
Number of fruits and vegetable eaten (per day)	3.8 (4.6)
Number of sugar-sweetened beverage consumed (per week)	14.8 (18.3)
Body mass index (pounds/inches^2^)	28.6 (7.1)

*^a^Participants who reported no missing data on any of the variables were included in the bivariate analyses*.

Most participants (86.7%) reported shopping at a supermarket as their usual source for groceries. Very few reported a corner store or a farmer’s market. The most frequently reported travel modes to the store included car (59.9%), walking (18.1%), and public transportation (10.8%). The mean distance to the nearest grocery store was 2.5 miles (SD = 4.1). The median distance was 1 mile, with 42.1% of people reporting living more than a mile from the nearest grocery store and 75.9% of people living more than 0.5 miles. The mean travel time to reach the nearest grocery store was 10.2 min (SD = 10.6). The median travel time was 5 min. While travel times ranged from 0 to 120 min, most reported traveling 5 min or less (50.3%) or 6–10 min (26.8%).

The average number of fruits and vegetables consumed per day was 3.8 (SD = 4.6). The median number was 2.1 and most people (77%) ate fewer than five fruits and vegetables per day. The average number of SSBs consumed per week was 14.8 (SD = 18.3). The median number consumed was 8. Over half (57.4%) reported consuming at least one SSB per day, while 32.1% reported weekly consumption, and 10% reported never consuming them. Mean BMI was 28.6 (SD = 7.1) and ranged from 17.9 to 50.5. About one-third (34.5%) of participants was classified as obese, a third (31.1%) was classified as overweight, and the final third (34.4%) was classified as having normal weight.

Distance and time to the grocery store were moderately correlated (ρ = 0.3209). Time spent traveling to the grocery store differed significantly by mode of travel (*p* < 0.00001), with public transportation being associated with the longest travel time, and car being associated with the shortest. Time spent traveling to the grocery store did not significantly differ by the usual type of store accessed (*p* = 0.1257) (Table 2).

**Table 2:** **Relationship between self-reported travel time to the nearest grocery store, travel mode, and usual grocery source among LA HANES participants, 2012 (*N* = 1,318)^a^**.

	Travel time to nearest grocery store (in minutes)	ANOVA *p*-value
	Mean (SD)	
**Travel mode**
Car	8.4 (9.1)	<0.00001
Public transportation	20.1 (15.0)	
Walk	9.6 (8.0)	
Bicycle/skateboard	11.2 (8.6)	
Multiple modes	11.3 (11.1)	
**Usual grocery source**
Supermarket	9.9 (10.3)	0.1257
Corner store	10.4 (6.7)	
Farmers market	13.0 (10.0)	
Other	16 (13.9)	
Multiple places	11.9 (14.4)	

*^a^Participants who reported no missing data on any of the variables were included in the analyses*.

In the multivariable models, neither distance nor time to the nearest grocery store was significantly associated with fruit and vegetable intake, SSB consumption, or BMI. Many of the control variables were significantly associated with the outcomes across models (Table 3). Use of categorical predictors and outcomes did not substantively change the results.

**Table 3:** **Multivariable regression models of the relationship between self-reported access to the grocery store and fruit and vegetable intake, sugar-sweetened beverage consumption, and body mass index among LA HANES participants, 2012 (*N* = 1,318)^a^**.

	Model 1: fruit and vegetable intake		Model 2: sugar-sweetened beverage consumption		Model 3: body mass index^b^	
	Rate ratio (95% confidence interval)^c^		Rate ratio (95% confidence interval)^c^		Coefficient (95% confidence interval)^d^	
**Food environment**
Miles from home to nearest grocery store	0.995 (0.981,1.009)	****	1.012 (0.996, 1.038)	****	− 0.023 (− 0.113, 0.067)	****
Travel time to nearest grocery store	****	1.005 (1.000, 1.010)	****	1.003 (0.997, 1.009)	****	0.007 (− 0.028, 0.043)
**Control variables**
Gender (ref: female)	0.895 (0.803,0.999)*	0.902 (0.809, 1.006)	1.281 (1.139, 1.441)***	1.281 (1.138, 1.442)***	− 2.263 (− 3.001, − 1.525)***	− 2.244 (− 2.980, − 1.509)***
Race/ethnicity (ref: African-American)
Latino	0.957 (0.837,1.094)	0.965 (0.845, 1.103)	0.625 (0.543, 0.723)***	0.623 (0.540, 0.718)***	0.190 (− 0.708, 1.089)	0.202 (− 0.696, 1.100)
White	0.763 (0.636,0.915)***	0.776 (0.647, 0.930)***	0.586 (0.483, 0.710)***	0.580 (0.478, 0.703)***	− 1.950 (− 3.162, − 0.737)***	− 1.918 (− 3.130, − 0.705)**
Other	1.058 (0.886,1.263)	1.059 (0.887, 1.264)	0.571 (0.470, 0.694)***	0.572 (0.471, 0.695)***	− 2.469 (− 3.680, − 1.258)***	− 2.461 (− 3.672, − 1.249)***
Education (ref: less than high school)
High-school graduate	1.021 (0.852,1.225)	1.039 (0.866, 1.246)	0.860 (0.708, 1.044)	0.886 (0.713, 1.053)	− 0.899 (− 2.132, 0.334)	− 0.871 (− 2.105, 0.363)
Some college	0.948 (0.793,1.110)	0.957 (0.808, 1.132)	0.748 (0.627, 0.892)***	0.753 (0.631, 0.898)***	− 0.776 (− 1.910, 0.358)	− 0.738 (− 1.874, 0.399)
College graduate	0.919 (0.755,1.119)	0.946 (0.776, 1.152)	0.518 (0.422, 0.637)***	0.523 (0.425, 0.644)***	− 1.271 (− 2.598, 0.057)	− 1.212 (− 2.545, 0.121)
Postgraduate/professional degree	1.306 (0.987,1.726)	1.337 (1.011, 1.768)	0.290 (0.213, 0.396)***	0.294 (0.215, 0.403)***	− 2.516 (− 4.447, − 0.585)*	− 2.456 (− 4.390, − 0.522)*
Age^b^	1.006 (1.002,1.011)***	1.006 (1.001, 1.010)***	0.988 (0.983, 0.992)***	0.987 (0.983, 0.992)***	0.111 (0.080, 0.140)***	0.109 (0.079, 0.139)***

*^a^Participants who reported no missing data on any of the variables were included in the multivariable analyses*.

*^b^Continuous variable*.

*^c^Negative binomial regression model*.

*^d^Linear regression model*.

****Covariate was excluded from the model.

*p < 0.05, **p < 0.01, ***p < 0.001.

Stratified analysis showed some similar trends across models. Consistently, among those who reported driving to the store, greater time and distance from the store was associated with eating more fruits and vegetables, for example, the rate ratio of the negative binominal regression model of time on total fruit and vegetable consumption (IRR = 1.008, 95% CI = 1.001, 1.015), approaching statistical significance (*p* = 0.04). Among those who used active transportation, greater time and distance from the store was associated with greater BMI, for example, the odds of being obese were 1.066 times as great (95% CI = 1.020,1.116) for each additional minute individuals lived from the store (*p* = 0.005), after controlling for other factors in the model.

## Discussion

Neither distance nor time to the nearest grocery store was significantly associated with fruit and vegetable intake, SSB consumption, or BMI across models. The results were robust to the specification of the model, including the use of continuous or categorical independent and dependent variables and stratified analysis. Previous literature has found mixed results on the association between elements of the food environment, eating behaviors, and health outcomes (11). The present study findings align with studies, which failed to find significant associations between distance to food outlets and dietary intake and BMI (12, 20).

There are many potential reasons for the null findings. First, Los Angeles County is considered to be a car-centric jurisdiction. In our sample, a majority of participants, even though they were low-income, reported using a car to get to the grocery store. This percentage is larger than those reported by other studies that have assessed travel mode to usual food source among similar populations (21). The increased reliance on car travel to access grocery outlets may lessen the importance of physical distance in determining food purchasing and consumption patterns (12). Although not consistent, results of our stratified analyses suggest that greater time and distance from the grocery store was associated with higher BMI among those who walked or biked to the store. There is support in the literature for this notion. The mitigating effects of car ownership have been observed in research examining the association between health outcomes and fast food density, wherein concentration of fast food restaurants was associated with higher BMI only in residents who lacked access to a car (22).

Second, access to grocery stores is frequently associated with increased access to food outlets generally, including fast food restaurants. In a recent study by Hattori and colleagues (12), low-income individuals were found to have greater access to all types of food outlets, as compared to their more affluent counterparts. Thus, disadvantaged communities have been described as “food swamps” – that is, they often contain a plethora of retail food establishments that sell unhealthy foods (23). Given these associations, the indicator “distance from the grocery store” may serve as a proxy for measuring general food access that also reflects the density of retail food venues that sell unhealthy products.

Results of this study support the need for a multi-dimensional approach when studying the relationship between food stores and health outcomes. In general, few studies have examined multiple dimensions of access simultaneously (24), such as within-store availability of healthy food (20), variety of retail options (25), or affordability, quality, and cultural acceptability in addition to proximity (26). As all of these factors are likely to impact consumption patterns, a more comprehensive approach to study the relationship between food environments and eating behaviors should measure access as well as factors related to food quality, price, and promotional environment. At minimum, indices that consider the presence of healthy and unhealthy foods outlets (27–29) would provide a more holistic appraisal of the settings in which people make food choices.

Looking beyond the use of cross-sectional data, for example, quasi-experimental designs should be considered to examine how the retail food environment affects diet and health. Several such studies have been conducted in the United Kingdom, with mixed results (30, 31). In the U.S., growing political momentum has enabled many full-service grocery stores to open in disadvantaged neighborhoods, providing an opportunity to further study environmental factors that can influence food access and food selection. Capitalizing on such momentum, future studies can help elucidate how changes in the immediate food environment may differentially affect the diet of underserved populations.

### Limitations

The present study provides insights on the association between self-reported food environment, fruit and vegetable intake, sugar-sweetened beverage consumption, and obesity. Despite its strengths, the study has a number of limitations. First, we relied on self-reported measures of time and distance to the nearest grocery store. While these measures provide an important perspective on access, the extent to which participants’ subjective categorization of retail outlets and distance represents a valid measure of food access is unclear. While over half of the people in the sample reported living more than a mile from the nearest grocery store, many may not live in a true “food desert.” According the USDA, very few census tracts in Los Angeles County meet the two criteria for living in a food desert (9). Our measures may not have been sensitive enough to identify people for whom food access is a true problem. Also, we asked people to identify the grocery store nearest to their home, which may not be the store at which they usually shop. Second, because the data were collected in public health centers, survey participants may have tended to over-report fruit and vegetable consumption and underreport SSB consumption as a result of social desirability bias. Additionally, the less than perfect response rate may have resulted in selection bias. Third, only two dimensions of healthy eating were assessed in the study: fruit and vegetable intake and sugar-sweetened beverage consumption. Lastly, because the study sample was not a true random sample of all LAC residents, the findings may not be generalizable to the general adult population in the region. None-the-less, the sample does represent the low-income segment of the LAC population that was targeted by recent obesity prevention efforts in the jurisdiction.

## Conclusion

Study findings failed to demonstrate a significant association between access to grocery stores, fruit and vegetable intake, sugar-sweetened beverage consumption, and obesity, suggesting that improving access to grocery stores as a standalone strategy to research and intervene against the obesity epidemic may not be sufficient. However, to misinterpret this null finding and discount the utility of the approach would be misleading. Rather, the results of the study, along with previous work, suggest that changing eating behaviors may require a multi-dimensional approach to conceptualizing the food environment. Such an approach could lead to more discernable impacts by considering the complexity of food environments and including synergistic measures and strategies that increase access to healthy, affordable options in multiple venues as well as educate and empower residents of these low-income communities to eat more healthfully.

## Author Contributions

Lauren Nichol Gase developed the conceptual framework for the analysis and led the data analysis. She drafted the primary text of the manuscript. Amelia Rose DeFosset provided technical assistance, including background research, consultation, and text revision. Lisa V. Smith led LA HANES data collection and provided technical assistance for data analysis. Tony Kuo designed the original LA HANES study; he consulted on the analysis plan and manuscript development. All authors have reviewed and approved the final version of the article.

## Conflict of Interest Statement

The authors report no conflicts of interest. The findings and conclusions in this article are those of the authors and do not necessarily represent the views or the official position of the Los Angeles County Department of Public Health.
